# Tone Mapping Operator for High Dynamic Range Images Based on Modified iCAM06

**DOI:** 10.3390/s23052516

**Published:** 2023-02-24

**Authors:** Yumei Li, Ningfang Liao, Wenmin Wu, Chenyang Deng, Yasheng Li, Qiumei Fan, Chuanjie Liu

**Affiliations:** 1 National Professional Laboratory of Color Science and Engineering, School of Optoelectronics, Beijing Institute of Technology, Beijing 100081, China; 2 Department of Chemical and Materials Engineering, University of Alberta, Edmonton, AB T6G 2V4, Canada

**Keywords:** HDR image, tone mapping, saturation compensation, image detail enhancement

## Abstract

This study attempted to solve the problem of conventional standard display devices encountering difficulties in displaying high dynamic range (HDR) images by proposing a modified tone-mapping operator (TMO) based on the image color appearance model (iCAM06). The proposed model, called iCAM06-m, combined iCAM06 and a multi-scale enhancement algorithm to correct the chroma of images by compensating for saturation and hue drift. Subsequently, a subjective evaluation experiment was conducted to assess iCAM06-m considering other three TMOs by rating the tone mapped images. Finally, the objective and subjective evaluation results were compared and analyzed. The results confirmed the better performance of the proposed iCAM06-m. Furthermore, the chroma compensation effectively alleviated the problem of saturation reduction and hue drift in iCAM06 for HDR image tone-mapping. In addition, the introduction of multi-scale decomposition enhanced the image details and sharpness. Thus, the proposed algorithm can overcome the shortcomings of other algorithms and is a good candidate for a general purpose TMO.

## 1. Introduction

High dynamic range (HDR) images have wide application prospects in the fields of medical imaging, aerospace remote sensing, and cross-media color reproduction because of their higher dynamic range, wider color gamut, and richer details [1,2,3,4,5]. The dynamic range of HDR images must be mapped to the range of display devices, which is called tone mapping [2]. The HDR of an image generally has a dynamic range, which is defined as the ratio between the highest and lowest luminance and is higher than three or four log10 units [3,4]. However, the dynamic range reproducible on traditional display devices is generally lower than these values and thus limits the best display of HDR images, resulting in dynamic range mismatch. Therefore, tone mapping operators (TMOs) must be researched and solved to faithfully display HDR images on conventional devices, which has attracted extensive attention and research [4,5,6].

Currently, HDR image TMOs mainly consist of global and local algorithms. Both primarily consider the compression of image luminance. However, the algorithms inspired by human color vision, such as the image color appearance model, while focus on the luminance compression and accurate reproduction of color [7]. Conventional global algorithms mainly include adaptive log transform compression, linear-gradient compression, histogram adjustment compression, and photographic reproduction [8,9,10]. Other new algorithms based on traditional algorithms have also been researched. Lee et al. [6] proposed a global tone-mapping operator based on a new asymmetric sigmoid curve to enhance global contrast. Based on the luminance histogram, Yang and Khan et al. [9,11] presented efficient methods, by using a Gamma function and constructing a lookup table (LUT), to enhance visual details. Jung et al. [12] proposed a naturalness-preserved tone mapping by applying perceptual quantization (PQ). Global methods offer advantages, such as the ability to process the intensity of all pixels according to the same scale and simpler calculation. However, certain details are generally lost in the case of tone mapped images.

Local tone-mapping algorithms consider different perceptions of luminance in different image regions, and the local image contrast and detail information can be relatively enhanced [13]. Typical local algorithms include the retinex theory [13], bilateral filter [14], guided filter [15], and multi-scale edge-preserving decompositions [16]. Based on multi-scale retinex, Lu et al. [17] introduced guided filtering instead of Gaussian filtering to effectively preserve image details. Gu et al. [18] proposed a novel filter for local edge-preserving decomposition, based on the multi-scale edge-preserving decompositions proposed in ref. [19]. The filtered image contains local means everywhere and preserves local salient edges, which have three detail layers and one base layer. However, the different degrees of local processing results in the local algorithms exhibiting poor continuity of gradient and the “halo” artifacts in the tone mapped images [18].

Global and local tone-mapping algorithms mainly focus on the mapping of luminance and the preservation of image details, whereas the accurate reproduction of color is neglected. However, the image color appearance model considers both the nonlinear compression of image luminance (also known as luminance adaption) and the accurate reproduction of image color [19,20,21]. In 2002, Fairchild and Johnson proposed iCAM [19,20], and then applied and extended it to HDR image compression [21]. iCAM is an image appearance model that attempts to determine the perceptual response to spatially complex stimuli and was extended to the tone map of HDR images [14]. In 2007, Kuang and Fairchild revised the iCAM model, referred to as iCAM06, to render HDR images [7]. iCAM06 offers the advantage of combination with a fast bilateral filter [14,18], which decomposes the images into base and detail layers to maintain the edge details of the image. However, certain problems remain. In 2012, Chae et al. proposed a compensation method using the corrected channel gain function to ameliorate the problem of white point-shift. They aimed to correct hue shift in iCAM06 and achieved better performance [2]. In 2013, Kwon et al. proposed a new method to find global illuminant information to reduce the desaturation effect for iCAM06 in the HDR-image rendering process [22]. In 2019, Kwon et al. [23], proposed a global chromatic adaptation, based on the color appearance model (CAM02), to improve the desaturation effect in iCAM06. This method was a chromatic adaptation (CA)–tone compression (TC) decoupling method that reduced the interference between the CA and TC.

Based on the image color appearance model, HDR image tone-mapping operators consider the color interaction of adjacent pixels and the influence of surrounding light to match a real human visual attribute, which is more suitable for tone-mapping reproduction of HDR images. However, existing tone-mapping operators considering the image color appearance model, such as iCAM06, are plagued by problems such as hue drift, desaturation, and detail loss [2,22,23]. Therefore, this study proposed an improved algorithm based on iCAM06, which ameliorates the above shortcomings and ensures the display of pleasant tone-mapped images on traditional display devices.

## 2. Algorithm Method

### 2.1. Method Procedure

A modified algorithm, iCAM06-m, combining iCAM06 with the multi-scale local detail-preserving decomposition (MSD) method was proposed. It can compensate for image saturation and correct hue drift.

**Figure 1:**
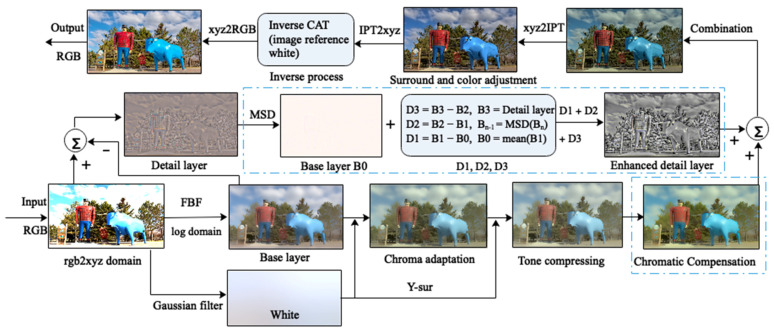
Flowchart of the modified algorithm for HDR images tone-mapping. The FBF was performed in the log domain, and then the images were converted to the XYZ space. All the images displayed are in the RGB space. The “White” was the adapted image, which is an extremely blurred image. The “Y-sur” is the luminance channel of “White” denoting the surrounding luminance. In MSD, B0 with no gradient was discarded, and the enhanced detail layer was the sum of three details: D1, D2, and D3. The calculation can refer to the following.

The procedure for iCAM06-m is shown in Figure 1. First, the image was decomposed into detail and base layers using fast bilateral filtering (FBF). Color adaptation and nonlinear compression were performed in the image base layer, followed by chroma compensation. The detail layer, including the luminance and chrominance components, preserved the image details. Further, in the improved algorithm, the MSD method [18,24] was added to the detail layer to enhance sharpness. Thereafter, the base and detail layers were combined and transferred into the IPT to adjust the surrounding illuminance and colorfulness. Finally, the compressed HDR image was output by inverse calculation and displayed on the conventional monitors.

### 2.2. iCAM06

The iCAM06 model [7] was proposed based on iCAM [20] for HDR-image tone mapping, which yields superior results owing to its ability to decompose images into base and detail layers. The base layer was obtained by using an FBF. Each image pixel was weighted by the product of Gaussian filtering in the spatial domain and another Gaussian filtering in the intensity domain. The detail layer was obtained by subtracting the base image from the original image in the log domain. Subsequently, they were converted to the XYZ space for the following process.

The base layer can be obtained using the following equation. The filtering calculation of central pixel q can be expressed as Equation (1):

(1) $$J_{out}=\frac{1}{W}\sum_{p,q\in S}f\left(\left|\left|p-q\right|\right|\right)g\left(I_{p}-I_{q}\right)I_{p}$$

with

$$W=\sum_{p,q\in S}f\left(\left|\left|p-q\right|\right|\right)g\left(I_{p}-I_{q}\right)$$

where *p* and *q* denote the locations of the pixels and the central pixel, respectively; *s* represents all pixel positions in the filtering window; *I* is the subsampling image of the input image; *W* normalizes the sum of the weights; *f*(*X*) is the space-domain Gaussian filtering with the kernel scale set to a practical value of 2% of the image size; *g(X)* is the intensity-domain filtering with its scale set to a constant value of 0.35; and *J* is the filtered image. 

In the base layer, chroma adaptation was performed in the cone response space, RGB, to adapt to the color component. Subsequently, TC was performed in the physiological cone response space $R^{\prime }G^{\prime }B^{\prime }$. The compression process is as follows:

(2) $$\left[\begin{array}{c} R^{\prime }\\ G^{\prime }\\ B^{\prime } \end{array}\right]=M_{HPE}M_{CAT02}^{-1}\left[\begin{array}{c} R_{c}\\ G_{c}\\ B_{c} \end{array}\right];$$

(3) $$R_{a}^{\prime }=\mathrm{sign}\left(R^{\prime }\right)\frac{400{\left(F_{L}R^{\prime }/Y_{Low}\right)}^{p}}{27.13+{\left(F_{L}R^{\prime }/Y_{Low2}\right)}^{p}}+0.1$$

(4) $$G_{a}^{\prime }=\mathrm{sign}\left(G^{\prime }\right)\frac{400{\left(F_{L}G^{\prime }/Y_{Low}\right)}^{p}}{27.13+{\left(F_{L}G^{\prime }/Y_{Low}\right)}^{p}}+0.1$$

(5) $$B_{a}^{\prime }=\mathrm{sign}\left(B^{\prime }\right)\frac{400{\left(F_{L}B^{\prime }/Y_{Low}\right)}^{p}}{27.13+{\left(F_{L}B^{\prime }/Y_{Low}\right)}^{p}}+0.1$$

(6) $$\left[\begin{array}{c} R_{TC}\\ G_{TC}\\ B_{TC} \end{array}\right]=\left[\begin{array}{c} R_{a}^{\prime }\\ G_{a}^{\prime }\\ B_{a}^{\prime } \end{array}\right]+A_{s};$$

where $R_{c}G_{c}B_{c}$ is the value of the CA obtained from XYZ; $M_{CAT02}^{-1}$ is the inverse transformation matrix of the cone response space; $M_{HPE}$ is the transformation matrix of the physiological cone response; $F_{L}$ is the adaption factor of luminance level, which is the function of the luminance channel of reference white; $Y_{Low}$ is the relative luminance image, also called surround luminance; p is the compression index from 0.6 to 0.85; $R_{TC}G_{TC}B_{TC}$ is the final TC image; and $R_{a}^{\prime }G_{a}^{\prime }B_{a}^{\prime }$ and $A_{s}$ are the TC of physiological cone response and rod response, respectively. The specific algorithm is referred to as the iCAM06 model [7].

In the base layer, following CA, color distortion occurs because each XYZ channel has a different intensity value [23]. Further, the detailed layer of iCAM06 includes the chrominance and luminance components. The recombined image of the base and detail layers affects the color distortion, which includes desaturation and hue drift. Furthermore, certain details of the base layer after CA and TC were lost. Therefore, in the improved algorithm iCAM06-m, the compensation chroma method by increasing saturation and correcting hue drift was considered. In addition, multi-scale local detail-preservation decomposition was applied to the detail layer, and the detail layer retained only the luminance component.

### 2.3. Multi-Scale Enhancement

The multi-scale local edge-preserving decomposition (MSD) was applied in iCAM06 in the detail layer to enhance the details [18,24]. Because low-pass filtering always causes a significant halo, the improved MSD decomposed the image into one base layer and three detail layers to avoid an artificial halo [18]. The improved MSD is based on the following three assumptions:

(1) The base layer remains local means in each local window;
(2) All scale’s salient details are relatively large gradients in every local window;
(3) The gradient information in the detail layer is non-zero everywhere.

Based on assumptions (1) and (2), the filtered base layer contained smooth local information and salient details, obtained progressively by calculating the local approximate means, instead of Gaussian filtering. The constraint conditions are as follows:

(7) $$\sum_{i\in \varphi }{\left(I_{i}-B_{i}\right)}^{2}+\frac{\alpha }{|\nabla I{|}^{\beta }}|\nabla B_{i}{|}^{2}\leq \varepsilon$$

(8) $$B_{i}=a_{\varphi i}I_{i}+b_{\varphi i},\;\;\;\;i\in \varphi$$

(9) $$B_{\varphi }=\frac{1}{N}\sum_{i\in \varphi }B_{i}\approx \bar{a_{\varphi }}I_{j}+\bar{b_{\varphi }}$$

In the first part of Equation (7), $B_{i}$ represents the filtered pixels and the second part is the gradient in every local window. α and β maintain a balance between the two terms and render the filtered $B_{i}$ as close to I as possible. $B_{i}$ can be considered as a linear function of $I_{i}$. When the cost function achieves the minimum ε, the values of $a_{\varphi i}$ and $b_{\varphi i}$ are the optimal solutions to Equation (8). In the local window, $B_{\varphi }$ is equal to the mean of the sum $B_{i}$. If $I_{j}$ represents the central pixel, $B_{\varphi }$ can be obtained approximately using $I_{j}$ in Equation (9). 

Based on assumption (3), the detail layers were obtained from the difference between the two recently filtered base layers. The salient edges depend on the size of the filtered local window. The process of the detail layers is as follows:

(10) $$B_{n-1}=MSD\left(B_{n}\right),\;n=m:-1:2,\;m=3,\;B_{m}=I$$

(11) $$D_{n}=B_{n}-B_{n-1}$$

The detail enhancement function is abbreviated as MSD. After decomposition, the image can be described using Equation (12). The base layer $B_{0}$, was obtained considering the mean of $B_{1}$, which is a smooth and uniform image with no gradient and will be discarded.

(12) $$I=B_{0}+D_{1}+D_{2}+D_{3}$$

(13) $$D_{i}=\frac{2}{\pi }\arctan(20\times D_{i})$$

(14) $$Out_{p}={\left(\frac{In_{p}}{I}\right)}^{\alpha }\sum_{i=1}^{3}\beta_{i}D_{i},\;\;p=r,g,b$$

The output image was accumulated in three detail layers and can be obtained using Equation (14). α can adjust the image saturation, which is in the range of 0.5–0.9. Further, β is the coefficient of the detail layer; $\beta_{1}=0.5,\;\beta_{2}=\beta_{3}=1$. The coefficient can adjust the degree of salient details in the images. 

The three detail layers at different scales were non-zero gradient salient edges, and their energy was remapped to enhance minor deviations around zero and compress large ones. Therefore, the accumulation results of the three detail layers possessed more detail than the original image.

### 2.4. Chroma Compensation

A chroma correction method was proposed and applied to the base layer. The CIELab color space, rather than the IPT, was used to determine whether it was suitable for HDR image correction and display. Both can be interconverted, L=100×I, a=150×P, b=150×T [25]. First, the chroma and hue angle of the original and tone mapped images were calculated and recorded as “before” and “after”, respectively. The chroma and hue angle of the tone- mapped image can be calculated using Equations (15) and (16):

(15) $$C_{after}=\sqrt{a_{after}^{2}+b_{after}^{2}}$$

(16) $$\theta_{after}=\mathrm{artan}\left(\frac{b_{after}}{a_{after}}\right)$$

where $C_{after}\;$ and $\theta_{after}$ denote the chroma and hue angle of the tone mapped image; *a* and *b* represent the chromaticity coordinates in the Lab color space, respectively. Assuming that the chromaticity of the image is$\;C_{befor}$, the chroma compensation factor *β* and hue angle compensation value Δ*θ* can be calculated as follows:

(17) $$\beta =\frac{C_{before}}{C_{after}}$$

(18) $$\theta_{before}=\mathrm{artan}\left(\frac{b_{befor}}{a_{befor}}\right)$$

(19) $$\Delta \theta =\theta_{before}-\theta_{after}\;$$

Finally, the compensated chroma $C_{comp}$ and corrected hue angle $\theta_{comp}$ were calculated, and the chromaticity coordinates of a and b were obtained using $C_{comp}$ and $\theta_{comp}$. In Equations (20) and (21), the coefficients α=λ×β and $\Delta \theta_{c}=\Delta \theta +\varepsilon$, where the coefficients λ and ε adjust the chroma balance for optimal visual effects:

(20) $$C_{comp}=\alpha C_{after}$$

(21) $$\theta_{comp}=\theta_{after}+\Delta \theta_{c}$$

(22) $$a_{comp}=C_{comp}\cos\left(\theta_{comp}\right)$$

(23) $$b_{comp}=C_{comp}\sin\left(\theta_{comp}\right)$$

The chroma compensation and hue correction were performed after tone mapping in the base layer, and after the combination of the detail and base layers, the chroma was further corrected for the adjustment of surroundings and illuminance in the IPT color space. The combination of both can produce a better compensation effect.

## 3. Experimentation 

In the subjective evaluation experiment, the images processed by iCAM06-m were compared with three other tone-mapping operators (TMOs), specifically iCAM06 [7], guided filtering (GF) [15,26], and the MSD algorithm [16,18]. Psychophysics evaluation methods are divided into reference and no-reference comparative methods [27,28]. The former method is generally compared with high-quality images or realistic scenes, whereas the latter method is generally compared with the memory image. In this study, the no reference comparative and categorical judgment methods [28,29] were adopted to quantify and evaluate the tone mapped images displayed on the screen.

### 3.1. Stimuli and Apparatus

In the experiment, the HDR images after tone mapping were presented on an HP liquid crystal monitor (HP24MQ,59983704055), with 2560 × 1440 pixels, D65 white point, and peak luminance of 300 cd/m^2^, which is a traditional display device. The display device was calibrated and turned on for 30 min before the experiment [27,28], satisfying the equipment required for psychophysical experimental tests.

Ledda et al. [30] and Cadik et al. [31] compared various TMOs, including iCAM06 and other TMOs, and their study showed contradictions in ranking caused by the selection of various attributes and scenarios of images [32]. Therefore, the HDR images were selected from six different scenes representing a typical environment, including indoor and outdoor scenery, landscapes, and specific objects. These are all representative images used to evaluate the TMOs for tone mapped images [28,32]. Their format was radiance RGBE. The image names were Paul Bunyan, Great lounge, Doll, Snowman, Yosemite, and Tinterna and had dynamic ranges of 5.3, 8.3, 5.4, 7.23, 6.4, and 3.3, respectively (according to the formula: DR=log10(max/min)) [3,4].

### 3.2. Experimental Scheme of the Subjective Evaluation

The experiment was conducted in a dark room, where the illuminance was 0.4 lux measured by the illumination photometer of SPIC 300 (Everfine Corp., Hangzhou, Zhejiang, China) Then, five gender-balanced and color-normal observers (with an average age of 27) evaluated the images [33]. They were all naive to the imaging experiment and experimental purposes [32], complied with ITU_R subjective evaluation standards [27,28], and were informed of the evaluation content and criteria to avoid the influence of personal factors on the experimental results.

**Figure 2:**
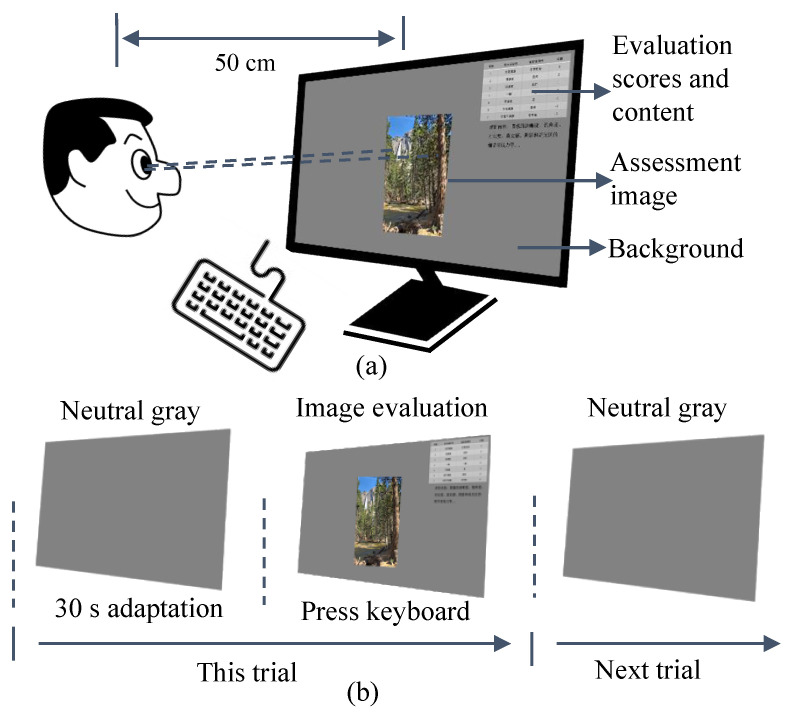
Diagram of the experimental procedure for subjective visual evaluation. (**a**) Tone mapped images, and the neutral grey background (20% grey). The evaluation content is on the upper right corner of the screen. The observation distance was 50 cm, and the field of view was 5°. (**b**) Each trail included 30 s adaptation [27], and then the images were evaluated by pressing the numbers on the keyboard.

From the previous psychophysical evaluation study for HDR tone-mapped images [27,28,29,30,31,32,33,34], Kuang et al. [29] and Drago et al. [33] used the method of no reference comparison with pairwise comparative judgement. Luo et al. [27] adopted a psychophysical categorical judgment method. In this study, no-reference HDR scenes and categorical judgment were performed [28,33]. The experimental setup and procedure for the subjective evaluation are shown in Figure 2. The corresponding evaluation levels were divided into seven categories from −3 to 3 quality levels, based on the degree to which the observer liked the compressed images (called the index of image preference). When evaluating, the tone-mapped image attributes, in terms of definition, saturation, contrast, details in shadows and highlights, and global appearance, were considered. Each observer performed three rounds of evaluation. The index of image preference was evaluated 72 times by each observer, for a total of 360 times.

## 4. Results and Discussion

**Figure 3:**
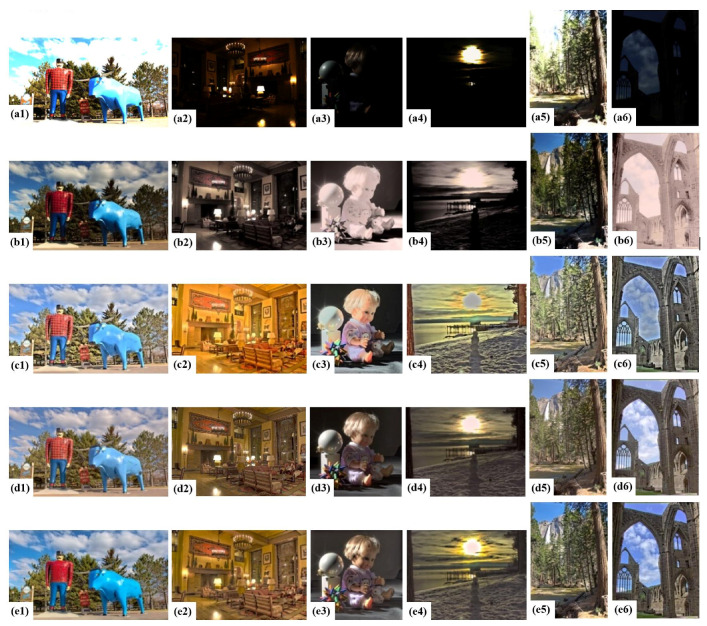
Images after tone mapping by four algorithms: (**a1**–**a6**) tone mapped images performed by linear normalization mapping method; (**b1**–**b6**) images processed by GF; (**c1**–**c6**) images operated using MSD; (**d1**–**d6**) images processed by iCAM06; and (**e1**–**e6**) images operated by the proposed algorithm, iCAM06-m.

The evaluation results of the proposed algorithm for the compression performance of HDR images were analyzed from both subjective and objective aspects. All the tone mapped images are shown in Figure 3.

Considering the proposed method combined iCAM06 and MSD, iCAM06 was compared with six methods and the MSD was compared with seven methods [29,33], the results of which all showed the performances of iCAM06 and MSD were optimal. Therefore, the proposed TMO was mainly compared with the two methods, which is simple and effective. The images in group (a1–a6) in Figure 3 were obtained using the linear normalization mapping method. Meanwhile, those of group (b1–b6), (c1–c6), (d1–d6), and (e1–e6) were obtained by guided filtering (GF), a Multi-scale local decomposition algorithm (MSD), iCAM06, and iCAM06-m, respectively.

**Figure 4:**
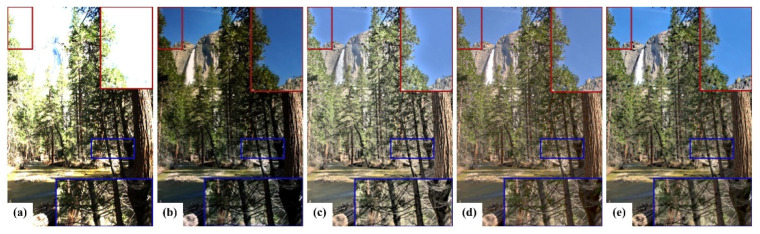
Tone mapped images. (**a**) Image performed by the linear normalization mapping method. (**b**) Image processed by GF. (**c**) Image operated MSD. (**d**) Image processed by iCAM06. (**e**) Images operated by iCAM06-m. The red enlarged area can check artifacts, and the blue enlarged area can check details.

It is evident that the image tone in group (b1–b6), processed by GF, is quite dark, and the shadow details are not clearly displayed. Moreover, color distortion is a serious issue. In contrast, the tone of the image mapped in group (c1–c6) is appropriate. The details in the highlights and shadows are better displayed. However, the images exhibit poor natural fidelity and “halo” artifacts, particularly at the edge of the blue sky in the red enlarged area in Figure 4c. The image tone in group (d1–d6) processed by iCAM06 is slightly dimmed, and the color is distorted in saturation and hue. Compared with the images in groups (c1–c6) and (d1–d6), in group (e1–e6), image saturation and hue are appropriate. In addition, the images have no “halo” artifacts and exhibit better continuity of gradient and natural fidelity, which retains the advantage of iCAM06. Thus, among the four TMOs, iCAM06-m exhibits the best performance for tone mapping and chroma correction.

From the above analysis, it can be concluded that the global appearance of tone mapped images depends on the appropriate image tone and on certain other local image attributes, including the reproduction of color, “halo” artifacts, and details. Compared with previous studies, this deduction was consistent with other results [27,28,31]. Ledda et al. compared six TMOs, and the results showed that the performance of iCAM06 was the best [30]. The MSD was compared with other seven methods by Gu et al., the results of which indicated that the performance of MSD was the best [30]. Furthermore, the performance of the proposed TMO was better than that of iCAM06 and MSD. Then, the performance of the proposed TMO was analyzed from subjective and objective aspects as follows.

### 4.1. Subjective Evaluation

**Figure 5:**
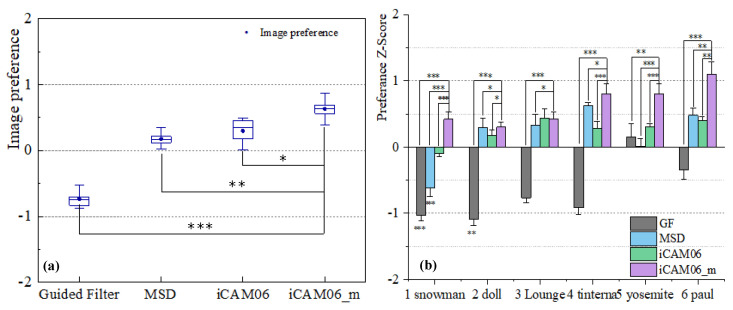
Subjective evaluation results. (**a**) Results of four algorithms for the compression performance in image preference. *T*-test was conducted for the difference between the proposed algorithm and other three TMOs. * indicates p<0.05, ** indicates p<0.01, *** indicates p<0.001. (**b**) Results of four algorithms of each image for the compression performance in image preference. The error bars denote 1 standard deviation. The significance difference between the proposed algorithm and other three TMOs, including each image, is calculated.

In the subjective experiment, owing to the different scoring benchmarks of each observer, all evaluation scores were processed with Z-scores, placing them on a unified ruler for comparison [35]. The subjective evaluation results of image preferences are shown in Figure 5. The evaluation preference scores are relative values. Figure 5a shows the evaluation results of four TMOs represented by a box-plot diagram. Figure 5b is the preference evaluation results of each image for the four algorithms about the image tone mapping performance.

In Figure 5a, the difference between the evaluation results of the proposed algorithm and other TMOs was tested using a *t*-test, and statistically significant differences were observed. This shows that the results of the subjective evaluation are reliable. Figure 5a shows that the proposed iCAM06-m exhibits a higher performance in terms of image preference. This is followed by iCAM06 and MSD (both relatively close), and the worst performer is GF. Figure 3b1–b6 shows the poor performance of GF, particularly in the image dim parts. Although the results of MSD and iCAM06 are relatively good, the tone mapped image cannot reproduce the image color appearance accurately. Compared to iCAM06, the image compressed by iCAM06-m exhibits improved chroma, hue angle, and details.

In Figure 5b, the statistical significance of the evaluation results in preference between iCAM06-m and other TMOs for each image is calculated. Almost every image exhibits significant differences between the proposed and other algorithms, thereby confirming the reliability of subjective assessment and the discrepancies in tone mapping performance for each TMO. Moreover, the results of the GF algorithm are the worst, and the MSD algorithm performs better in certain images, but not in others. In general, the compression performances of iCAM06 and iCAM06-m are better, and iCAM06-m is more stable. Furthermore, from Figure 3e1–e6 and Figure 5b, the iCAM06-m exhibits the better performance for images ‘Paul’, ‘Yosemite’, and ‘Tinterna’ in terms of image preference, which have high saturation and luminance.

### 4.2. Objective Evaluation

Objective image quality assessment indices (IQAIs) are simple and efficient for predicting the real perception of images by human vision. This study adopted the typical IQAIs, tone-mapped quality index (TMQI) [36], and the universal IQAIs, including image information entropy (IE) [37,38], image sharpness (IS) [39], and image chroma (IC) [40], to evaluate the tone mapped images [27]. The IQAIs can be calculated as follows:

(24) $$\mathrm{TMQI}=wS^{\alpha }\left(1-w\right)N^{\beta },\;0<w<1$$

(25) $$\mathrm{IE}=\sum_{i=0}^{255}-p_{i}log\left(p_{i}\right)$$

(26) $$\mathrm{IS}=\frac{1}{\left(R-1\right)\left(C-1\right)}\sum_{i=1}^{R-1}\sum_{j=1}^{C-1}\sqrt{\frac{{\left(x_{i,j}-x_{i+1,j}\right)}^{2}+{\left(x_{i,j}-x_{i,j+1}\right)}^{2}}{2}}$$

(27) $$\mathrm{IC}=\frac{1}{\left(R-1\right)\left(C-1\right)}\sqrt{a_{i,j}{}^{2}+b_{i,j}{}^{2}}$$

where w represents the proportion of structural fidelity *S* and natural retention *N*; α and β are the adjustment index of *S* and *N*, which are 0.304 and 0.708, respectively. The calculation of *S* and *N* can be referred to [36]. In Equation (25), the parameter i is the gray values of tone- mapped images, and $p_{i}$ represents the probability of i. In Equation (26), $x_{i,j}$ denotes the image gray value of different pixels, R and C are the numbers of image rows and columns. The $a_{i,j}$ and $b_{i,j}$ represent the image chroma in CIELAB space of the tone- mapped images.

**Figure 6:**
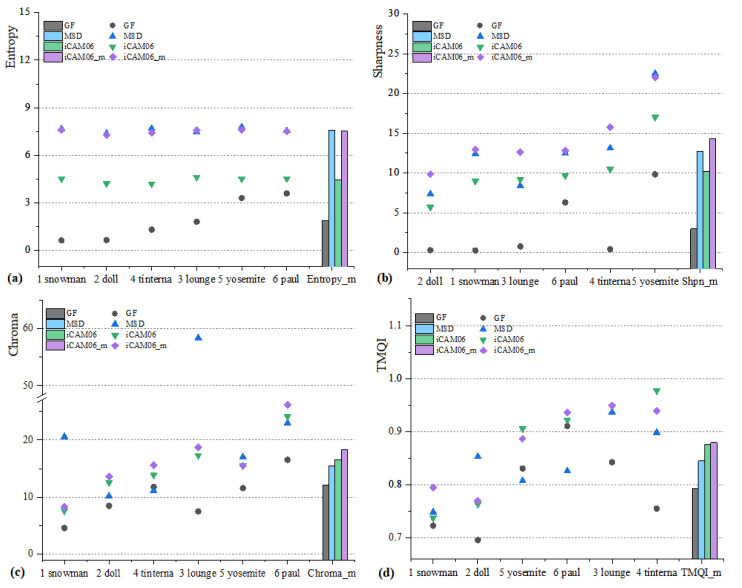
Assessment results of each image from 4 IQAIs. (**a**) The IE distribution of each image processed by 4 TMOs and the mean entropy of 4 TMOs. (**b**) The IS distribution of each image processed by 4 TMOs. (**c**) The IC distribution. (**d**) The TMQI distribution.

The IQAIs of the tone mapped images of the four TMOs are listed in Table 1. The IQAIs results of each image and mean performance of all images are shown in Figure 6. The IE distributions of each image processed by four TMOs are shown in Figure 6a, the histogram represents the average IE of all images for the four TMOs. Figure 6b shows the IS distributions of each image and the histogram is the mean IS of all images processed by four TMOs. Figure 6c,d were the IC and TMQI distribution, respectively.

The tone-mapped quality index (TMQI) [36] is an evaluation index that assesses the ability of tone mapped images to maintain the original structure and natural fidelity. The TMQI, TMQI-S, and TMQI-N represent the comprehensive index, structure, and natural fidelity, respectively. The larger the indices, the higher the structure and natural fidelity. As evident in Table 1, the TMQI performance of iCAM06-m was better than that of the other TMOs and was close to that of iCAM06, which can also be observed in Figure 6d. The MSD can cause artificial ‘halo’ to destroy the image structure fidelity. Thus, the proposed algorithm can keep the advantages of iCAM06 to maintain the original image structure and natural fidelity.

Image entropy (IE) [37,38] refers to the grey distribution information contained in the image. The greater the IE, the more uniform is the gray distribution of the image. From the IE values, the performances of iCAM06-m and MSD were similar. The method of MSD has a good ability to adjust the gray distribution in IE, but MSD is only applied in the detail layer of the image. When the detail layer and the base layer are combined, the IE is reduced in iCAM06-m. Figure 6a shows the IE of each image was relatively consistent between iCAM06-m and MSD, indicating that the gray distribution of iCAM06-m added MSD was significantly improved compared to that of iCAM06.

Image sharpness (IS) [39] is the ability to present minute details in images. From the values of IS in Table 1, it is evident that the iCAM06-m exhibited the best performance in preserving image details, followed by MSD, iCAM06, and GF, which can also be observed in Figure 6c. The results indicate that iCAM06-m has a better ability to enhance details than iCAM06. In colorimetry [40], the larger the chroma of images, the higher the image colorfulness, and the more pleasant visual perception experienced. As in Table 1, the chromas of iCAM06 and iCAM06-m are higher than that of MSD and GF, indicating the better performance of chroma prediction than that of other TMOs without considering the color vision model. Simultaneously, the image chroma tone-mapped by iCAM06-m is the highest, indicating that the chroma correction is effective.

Overall, iCAM06-m performs better in the four IQAs by combining the advantages of iCAM06 and MSD method and compensating the image chroma, especially when processing images with high saturation, high luminance and rich details, such as images ‘Paul’, ‘Yosemite’, ‘Tinterna’, and ‘Lounge’, which can be seen in Figure 3e1–e6 and Figure 6. The entropy (IE) and clarity (IS) performances of iCAM06-m are similar to those of MSD, indicating that the introduction of MSD into iCAM06 improved its ability. Further, about the chroma and TMQI, the performances of iCAM06-m and iCAM06 are similar and better; thus, it can be concluded that iCAM06-m can more accurately reproduce the image color appearance than iCAM06. The chroma compensation method corrected the hue drift and improved the saturation of tone mapped images.

## 5. Conclusions

The modified algorithm, iCAM06-m, combines iCAM06 and MSD, and compensates for the image chroma by correcting saturation and hue drift. The performance of iCAM06-m to predict the tone mapped images was evaluated from both objective and subjective aspects. The results of the subjective scores and objective IQAIs tended to be consistent and showed that iCAM06-m performed well and had satisfactory effects on tone mapped images.

In summary, the iCAM06-m improved the defects of iCAM06, including the loss of image details, desaturation, and hue drift, and combined the advantages of both iCAM06 and MSD. (1) The proposed chroma compensation algorithm in iCAM06-m improved the saturation and hue shift. (2) It inherited the stability of iCAM06 for tone mapping and retained the image color appearance, structure, and natural fidelity. (3) It preserves and enhanced the image details and image sharpness of MSD. Moreover, iCAM06-m based on iCAM06 can offered more advantages than other TMOs to predict the real image color appearance. Therefore, the tone mapped images obtained by iCAM06-m can accurately reproduce the image color appearance and provide more image details, indicating that iCAM06-m is a good candidate for a general purpose TMO.

## Figures and Tables

**Table 1:** Objective evaluation results of image *^a^*.

Mean	TMQI	TMQI-S	TMQI-N	IE	IS	IC
GF	0.793	0.7188	0.267	1.882	2.981	12.095
MSD	0.845	0.731	0.524	7.585	12.721	15.454
iCAM06	0.874	0.789	0.543	4.428	10.168	16.527
iCAM06_m	0.880	0.774	0.634	7.494	14.338	17.965

*^a^* Note. Because of the instability of the MSD for chroma prediction, as shown in Figure 6c, the mean chroma does not include image 1 and 3.

## Data Availability

Data will be made available on request.
